# Age and generational differences in anthropomorphism and trust in large language models

**DOI:** 10.3389/fcogn.2026.1824836

**Published:** 2026-07-20

**Authors:** Michelle Cohn, Mahima Pushkarna, Mark Díaz, Joscelin Cooper, Gbolahan O. Olanubi, Joseph M. Moran, Zion Mengesha, Courtney Heldreth

**Affiliations:** 1 Department of Linguistics, University of California, Davis, Davis, CA, United States; 2 Google Research, San Francisco, CA, United States; 3 Google DeepMind, Cambridge, MA, United States; 4 Google Research, New York, NY, United States; 5 Google, Cambridge, MA, United States; 6 Department of Anthropology, University of California, Los Angeles, Los Angeles, CA, United States; 7 Google Research, Kirkland, WA, United States

**Keywords:** age variation, anthropomorphism, human-AI interaction, large language models (LLMs), trust

## Abstract

**Introduction:**

As large language models (LLMs) increasingly mediate everyday information seeking, a fundamental question emerges: do people conceptualize these systems as social agents, and does this tendency vary by age?

**Methods:**

We investigated how individuals anthropomorphize and trust a pseudo-LLM, examining English-speaking adults from the United States (*n* = 1,485) across four generations: Gen Z (age 18–26), Millennials (age 27–42), Gen X (age 43–57), and Baby Boomers (age 58–77). We experimentally manipulated anthropomorphic cues (text versus text+speech; first-person “I” versus third-person “the system” framing).

**Results:**

Age and generational differences were observed: Gen Z (the youngest age group) showed consistently lower anthropomorphism, reduced trust ratings of the system, and lower ratings of accuracy for responses generated by the LLM. Anthropomorphic voice cues increased perceived human-likeness and accuracy uniformly across age groups. Qualitative analyses further revealed age and generational differences in how participants conceptualized the system.

**Discussion:**

Together, these findings suggest that age and generational differences shape how people attribute mind and agency to artificial systems.

## 1 Introduction

Increasingly, people use large language models (LLMs), such as OpenAI's ChatGPT^1^ and Google's Gemini^2^, to complete tasks (e.g., writing an email, summarizing a document, generating a story) (Wu et al., 2023). LLMs are a type of generative artificial intelligence (GenAI) that can produce responses that can remain contextually relevant to the user's input, can be indistinguishable from human-generated responses (Ariyaratne et al., 2023; Huschens et al., 2023). At the same time, LLMs have been shown to replicate the biases of their real-world training datasets (Acerbi and Stubbersfield, 2023; Dev et al., 2020; Gadiraju et al., 2023; Hofmann et al., 2024) and even generate fictitious data (“hallucinate”) in cases where training data is sparse (Zellers et al., 2019).

Thus, a central question facing users of LLMs is: how do I know what to trust? Research in social cognition suggests that humans routinely make inferences about the minds and intentions of others using available social cues (Adolphs, 2009; Frith and Frith, 2007). These processes also extend to human–computer interaction, leading people to attribute human-like characteristics, mental states, and agency to non-human systems (Gray and Wegner, 2012)—a process known as anthropomorphism (Epley et al., 2007; Fisher, 1991). Prior work has shown that how human-like a system seems shapes how much people trust it. For example, people trust robots, avatars, and conversational agents more if they display more human-like features (Hancock et al., 2011; Kim and Sundar, 2012; Rheu et al., 2021; Roesler et al., 2021; Seymour and Van Kleek, 2021; Waytz et al., 2014).

Importantly, LLM evaluations might not be uniform across individuals; prior work has shown age-related differences in anthropomorphism (Herrmann, 2023; Primanto and Amin, 2024) and trust (Belen Saglam et al., 2021; Pak et al., 2014; Rovira et al., 2019) for other types of systems (e.g., voice assistants, self-driving cars). Furthermore, age may influence how people integrate anthropomorphic cues. Indeed, prior work has shown that people combine multiple social signals, such as non-verbal and verbal cues, when inferring agency and trustworthiness of an AI system (Cohn et al., 2024; Hosseinpanah et al., 2018; Seeger and Heinzl, 2021; Waytz et al., 2014). Yet, to our knowledge, no prior work has examined whether integration of anthropomorphic cues in LLM evaluations varies across age groups.

The current study tests how adults of different ages evaluate the outputs of an LLM across a range of scenarios. Specifically, we test: (1) How do anthropomorphic cues shape evaluations of an LLM's output? (2) Does the influence of these cues vary as a function of age?

### 1.1 Anthropomorphism

Anthropomorphism, when a person attributes human-like qualities and behaviors to a real or imagined system (Epley et al., 2007; Waytz et al., 2010), is a common psychological response by people across a wide range of ages (Goldman and Poulin-Dubois, 2024; Kim and Sundar, 2012; Müller et al., 2018). Theories of technology equivalence posit that even when people know the system is not a human, they subconsciously apply behaviors from human-human interaction (Lee, 2008; Nass et al., 1997).

A number of studies have experimentally manipulated cues of human-likeness to explore their impact on anthropomorphism (Cohn et al., 2024; Gong, 2008; Moussawi and Benbunan-Fich, 2021; Qiu and Benbasat, 2005; Seeger and Heinzl, 2021; Torre et al., 2019). Some studies have manipulated a single cue at a time; for example, several studies have found that the presence of a voice, relative to a text-only condition, increases anthropomorphism and trust (Cohn et al., 2024; Moussawi and Benbunan-Fich, 2021; Qiu and Benbasat, 2005). Other approaches have tested multiple cues in concert; for example, Seeger and Heinzl (2021) had young adults (mean age = 21 years) interact with a customer service conversational agent in one of two conditions: a less human-like condition (where the system replied immediately, did not self-reference) and a more-human-like condition (where the system used personal pronouns, e.g., “I,” “me,” and responded with a delay). They found greater anthropomorphism and trust for the more human-like system with both cues.

Waytz et al. (2014) manipulated how young adults (mean age = 26 years) perceived an autonomous vehicle in one of three conditions: when driving themselves (baseline), when the vehicle drove autonomously (more human-like), and when the vehicle drove autonomously, had characteristics (a name and gender), and a pre-recorded human voice (most human-like). They found a cline of both anthropomorphism and trust: the least human-like vehicle (baseline) was the least anthropomorphized and least trusted. The self-driving car without human attributes was slightly more anthropomorphized and trusted. Finally, the self-driving car, “Iris,” was anthropomorphized and trusted the strongest.

### 1.2 Age differences in anthropomorphism

Anthropomorphism has also shown to vary based on a person's age. For example, Herrmann (2023) found that adults ages 54–72 were more likely to rate text-to-speech (TTS) voices as sounding like a human than younger adults (ages 20–38). In a comparison of Millennials (ages 29–39 years) and Generation Z (ages 17–28 years), Primanto and Amin (2024) found that Millennials rated a customer service chatbot as being more visually similar to a human, having higher empathy, and being more trustworthy, compared to a younger adult cohort, Generation Z. Comparing younger adults (ages 17–22), middle aged adults (ages 41–53), and older adults (ages 64–79), Xu et al. (2015) found that younger adults found a health robot to be “scary” and “unfriendly.”

Building on age-based differences in the tendency to anthropomorphize non-human agents, researchers have begun to explore how well people detect AI vs. human-generated content. Kreps et al. (2022) found that, in general, adults (mean age = 40 years) were largely incapable of distinguishing AI and human-generated text. Ramu et al. (2023) studied Generation Z participants (ages 13–23), and also found they were unable to discern between AI and human-authored text, although this was mediated by age and education level within the sample, where older and college-level participants were more accurate. Frank et al. (2024) compared participants aged 18–64, and found that German-speaking older adults were less likely to detect AI-generated audio than younger adults, but that there was no significant relationship between age and image or text detection accuracy.

### 1.3 Age differences in trust in technology

As with anthropomorphism, there is work showing differences in trust in technology by age (summarized in Table 1). On the one hand, there is some work suggesting that younger generations show stronger trust toward technology (Chan and Lee, 2023; Noah and Sethumadhavan, 2019).

**Table 1:** Overview of studies examining generational differences in trust.

Studies	Younger adults(s) finding (relative to older adults)
Chatbot (Primanto and Amin, 2024); health robot (Xu et al., 2015); AI-generated audio (Frank et al., 2024)	↓ Anthropomorphism
Voice assistants (Noah and Sethumadhavan, 2019); ChatBots storing information (Belen Saglam et al., 2021)	↑ Trust
Virtual assistant agent (Hosseinpanah et al., 2018); automation (Pak et al., 2017); self-driving cars (Rovira et al., 2019); clinician agent (Pak et al., 2014); customer service ChatBot (Primanto and Amin, 2024)	↓ Trust

Indeed, younger individuals (13–24 years) show stronger trust for voice assistants (e.g., Alexa, Siri, Cortana, and Google Assistant) compared to middle aged adults (40–60 years) (Noah and Sethumadhavan, 2019). Younger adults (ages 18–24 and 25–34) were also found to have stronger trust in chatbots storing their personal data, relative to middle age (ages 45–55) and older adults (ages 55+) (Belen Saglam et al., 2021). Additionally, Chan and Lee (2023) found that college-age adults were more likely to report using Generative AI (GenAI) (e.g., ChatGPT) more frequently than their teachers, and are more likely to report that GenAI can positively impact teaching and learning. The teacher cohort, on the other hand, were more likely to indicate they would fact-check and validate information from GenAI and were more likely to indicate GenAI has the potential to generate inaccurate information, as well as exhibit bias (Chan and Lee, 2023). Therefore, one prediction for the current study is that younger adults might readily trust knowledge from an LLM, compared to older adults.

On the other hand, other studies have shown the opposite pattern: older adults have shown to have stronger trust in technology than younger adults (e.g., self-driving cars, virtual agents, robots) (Pak et al., 2014, 2017; Rovira et al., 2019). For example, Pak et al. (2014) found that older adults (mean age 72 years) tended to trust a clinician agent more than younger adults (mean age 18 years). Degree of reliability appears to be a factor that differentially affects trust in technology for older and younger adults. Rovira et al. (2019) tested how younger (ages 18–51) and older adults (ages 65–87) rated trust in scenarios with self-driving cars, manipulating degree of risk and reliability. They found that both age groups showed higher ratings of trust for reliable cars, and lower trust for riskier situations. While there was no overall difference in trust by age group, they did see that younger adults were more sensitive to the reliability of the car (i.e., if there was a technology failure) and downgraded their trust more than older adults; but they did not see differences based on travel risk between the age groups.

Pak et al. (2017) found that young adults (mean age 19) and older adults (mean age = 71) varied in their level of trust for automated technology presented in a scenario, testing four domains (consumer electronics, banking, transportation, and health) and varying reliability of automation. They found that older adults showed higher trust for automation compared to students. Hosseinpanah et al. (2018) also found that older adults (mean age 66 years), relative to young adults (mean age 24 years), rated a virtual assistant agent exhibiting emotional non-verbal behavior as more empathic and trustworthy. Similarly, Vinichenko et al. (2022) found that college-age students in Slovakia and Russia were wary of historical information provided by AI. Therefore, an alternative prediction for the current study is that younger generations will show less reduced trust in the information provided by an LLM.

### 1.4 Current study

The current study investigates the effect of age on anthropomorphism and trust by adults for a pseudo-LLM system. One possible source of age-related variation is social cognition. Related work shows that adults differ in how they perceive and integrate social and emotional information across the lifespan (Blanchard-Fields and Abeles, 1996; Carstensen, 2021; Keightley et al., 2006). For example, socioemotional selectivity theory describes a relative shift in the importance of emotionally meaningful goals as individuals age, as well as increased attention to positive emotion and positively framed information (Carstensen, 2021). As a result, the use domain, the type of content produced, and its emotional framing could each play a role in how younger and older adults interpret and attend to anthropomorphic cues in technology. Consistent with this possibility, older adults perceive modern voice technology as more human-like than younger adults (Herrmann, 2023; Zellou et al., 2021). Additionally, there are age-based differences in cue integration. For example, Chin et al. (2024) found that older adults (ages 65–80) and middle aged adults (ages 50–64) responded differently to conversational style cues in a voice assistant. Older adults rated the systems more likeable overall and showed greater technology acceptance when the assistant used more informal language, while there was no effect of formality for the middle-aged adults. Consequently, anthropomorphic cues such as a human-like voice and use of the first-person “I” may differentially influence how younger and older adults evaluate the trustworthiness and reliability of LLM outputs.

One framework for probing age-related differences in trust and anthropomorphism comes from generational cohort theory (Okros, 2020; Twenge et al., 2015), which proposes that societal changes and historical events shape an individual's attitudes and values. Different generations were exposed to different forms of technological advancements, such as the internet and modern voice technology. For example, Generation Z (“Gen Z”) (born between 1997 and 2012) grew up with the internet and social media (Turner, 2015) and are considered digital natives (Prensky, 2001). Other age cohorts include Millennials (also referred to as Generation Y) (born between 1981 and 1996), Generation X (“Gen X”) (born from 1965 to 1980), and Baby Boomers (born from 1946 to 1964) (Dimock, 2019). To distinguish cohort-based effects from broader age-related patterns, we directly compare continuous age and generational cohort throughout our analyses.

Furthermore, while most prior work examining anthropomorphism and trust has focused on a single context, such as autonomous driving (Waytz et al., 2014), health care (Xiao et al., 2023), legal decisions (Kahr et al., 2023), and customer service (Primanto and Amin, 2024; Seeger and Heinzl, 2021), the current study examines how participants perceive information in five types of contexts that are common to voice technology interactions: cooking, career, travel, health disorders, and medications. These domains also vary in the perceived risks of inaccurate information. For example, cooking and travel represent relatively low-risk informational domains, whereas information about health disorders and medications could have greater consequences if inaccurate. Examining multiple domains allows us to test whether responses to anthropomorphic cues generalize across lower- and higher-risk contexts and whether weighting of these cues varies by age.

## 2 Methods

The current study analyzes data reported previously in Cohn et al. (2024), where we parametrically manipulated both the presence of a TTS voice and the language the system responded with: either “I” more human-like (as in Seeger and Heinzl, 2021) or “the system” (less human-like) in a sample of 2,165 participants from the United States. The present study re-examines a sample of this dataset, balanced across four generational cohorts. Specifically, we investigate whether adults of different ages differ in their overall perceptions of LLMs and the extent to which they integrate multiple anthropomorphic cues to shape their evaluations of the LLM. In addition, we incorporate qualitative analyses of participants' description of the system, providing insight into how people conceptualize and reason about LLMs across age groups.

### 2.1 Participants

Participants (*n* = 1,485; summarized in Table 2) consisted of adults from the United States who spoke English. All participants had prior experience with voice technology and all reported no hearing disorders. This sample included age groups that had a sample size of at least 350 participants (Gen Z, Millennial, Gen X, and Baby Boomer), excluding the Silent Generation. As there were more Millennials (*n* = 694) and Baby Boomers (*n* = 670) in the original cohort, we randomly selected a subset balanced across Voice and Grammatical Person conditions. Prior to data collection, the survey was reviewed by the Google Ethics Board. The following safety measures were taken to ensure the protection of participants: all participants were given the option to opt out of any sensitive questions (e.g., age, gender, race/ethnicity) and completed informed consent. No identifying information (e.g., name, address, etc.) was collected. All participants were compensated for their time.

**Table 2:** Participant demographics.

Participant demographics	Gen Z	Millennial	Gen X	Baby Boomer
Mean age (sd)	22.1 (2.5)	24.7 (4.3)	49.6 (4.7)	67.3 (5.2)
Age range	18–26	27–42	43–57	58–77
Gender				
Women	250	244	194	204
Men	98	131	183	175
Another gender	4	0	1	1
Race and ethnicity				
Asian	35	24	18	4
Black/African American	98	62	74	26
Indigenous/Native American	30	20	18	12
Hispanic/Latino	175	97	54	18
white	177	261	271	341
Distribution across conditions				
1SG + Speech + text	92	91	87	95
1SG + Text only	94	94	100	95
3SG + Speech + text	90	94	95	95
3SG + Text only	86	96	96	94
Total	352	375	378	380

### 2.2 Procedure

Participants followed the procedure as described in Cohn et al. (2024), completing the study online using Qualtrics. After an initial audio calibration to ensure they could hear the audio, participants were told that they would be interacting with a system that could “answer questions about work, health, and general facts.” Participants completed 20 experimental trials (order randomized) across five domains (health, medication, career, travel, and cooking) in a vignette-based design. For a full list of contexts, questions, and system responses, see Supplementary Table 1.

**Figure 1:**
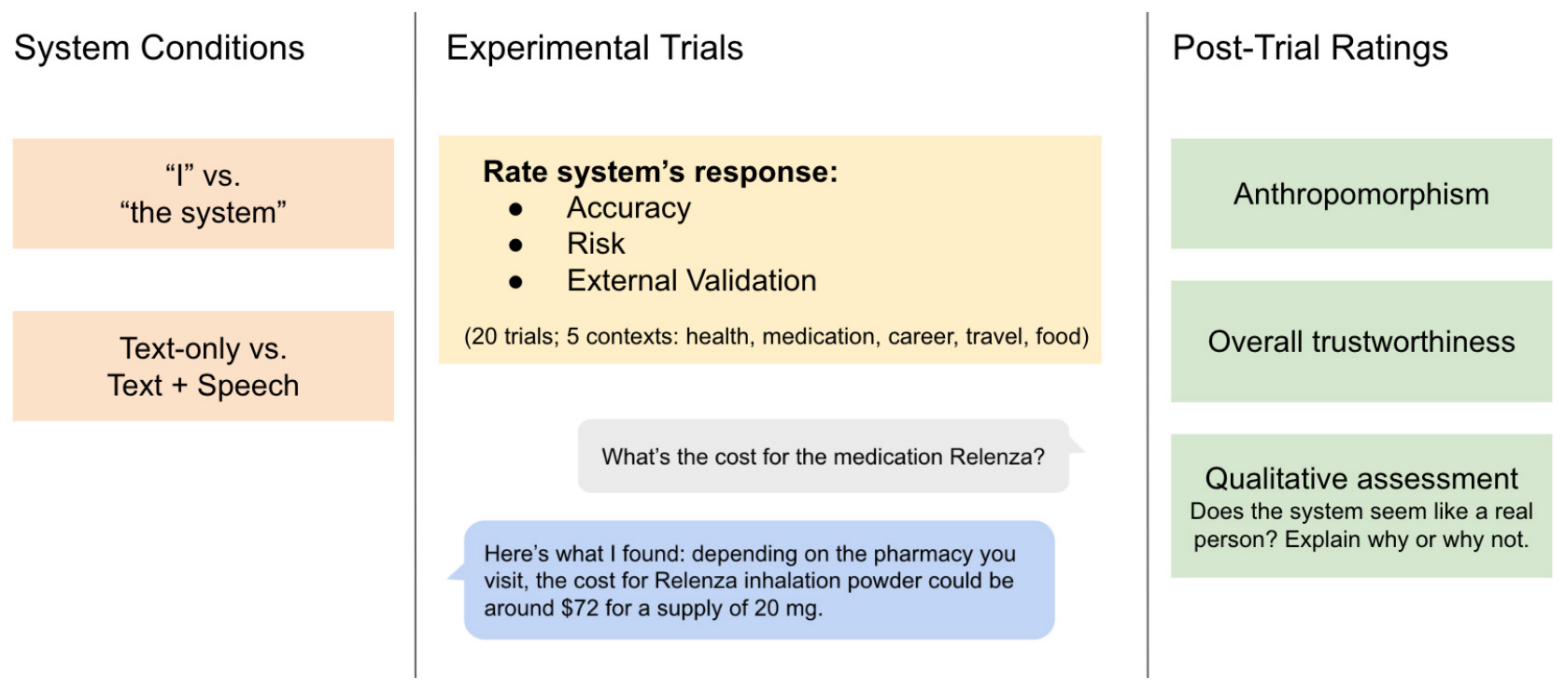
Procedure for the current study, adapted from Cohn et al. (2024). Participants completed the experiment in one of the pronoun conditions (“I” vs. “the system”) and one of the modality conditions (“text-only” vs. “text + speech”). Participants completed 20 experimental trials, where they rated the system's response on three dimensions: accuracy, risk, and likelihood to externally validate. After completing all of the experimental trials, participants rated the system's anthropomorphism, trustworthiness, and completed a qualitative assessment, where they provided a short answer response as to “Does the system seem like a real person? Explain why or why not.”

On each trial (example shown in Figure 1; full list of stimuli provided in the Supplementary Table 1), participants clicked “ask” to submit a pre-typed question to the system (e.g., “Should I take the medication Topamax if I'm already taking Tylenol?”) and after 500 ms, a sound effect indicated it had been submitted. Next, the system showed “...” (1,500 ms), which was replaced by the system's response, which varied according to the two anthropomorphic manipulations (between-subjects design):

- **“I” vs. “the system”**. For the grammatical person manipulations, the system either responded with “Here's what I found” or “Here's what the system found.”
- **Text vs. text** **+** **speech**: For text + speech, participants saw the text and additionally heard a text-to-speech (TTS) voice read the response aloud simultaneously. The TTS stimuli (provided in the Open Science Repository for the project)^3^ were generated using a neural TTS voice (US-English Studio-O^4^, female). The average fundamental frequency (perceived as pitch) was 224.28 Hz (sd = 6.16), consistent with an apparent female voice, while the average speaking rate was 4.26 syllables per second (sd = 0.27), consistent with a clear speaking style (Smiljanić and Bradlow, 2008). We generated the preambles (“Here's what I found,” “Here's what the system found”) separately from the answer content and then concatenated the audio files; this meant the preamble was acoustically identical across conditions. Sound files were amplitude normalized to 65 dB. We measured the total duration of the audio files for each trial (“Here's what I found” and “Here's what the system found”) and selected the longer of the two durations for each item (rounded to nearest second) (reported in the Supplementary material) and added an additional 3,500 ms to it to set a standardized interval before the response options appeared on screen.

After each trial, participants rated the system's response on three dimensions: accuracy, risk, and validation. Accuracy was rated on a 4-point Likert scale (Not at all accurate to Completely accurate). Perceived risk was rated on a 4-point scale (Not at all risky to Extremely risky). Participants also indicated whether they would validate the response with another source (No, Maybe, Yes).

After completing all 20 trials, participants rated the system's overall trustworthiness on a 5-point scale (Extremely untrustworthy to Extremely trustworthy). Emotional trust was assessed using four statements: the system is unbiased, honest, acts in the user's best interest, is knowledgeable, where participants indicated True or False (Benbasat and Wang, 2005; Wang and Benbasat, 2007). Participants additionally completed five anthropomorphism items from the Godspeed questionnaire (Bartneck et al., 2009): fake—natural, machine-like—human-like, unconscious—conscious, artificial—real, and incompetent—competent. All anthropomorphism items used 5-point semantic differential scales.

Next, they were asked an open-ended question: “Do you think the system is like a real person? Explain why or why not.” Finally, to confirm participants were all listening to the audio for the entire duration of the experiment, they completed a listening comprehension check. All participants passed the listening comprehension question.

## 3 Statistical analysis and results

In the following sections, we provide the analysis and results for Anthropomorphism (Section 3.1), ratings of trust of the system overall (Sections 3.2, 3.3), subdimensions of Anthropomorphism (Section 3.4), whether the system “seems like a real person” (Section 3.5), and trial-level ratings of trust (Section 3.6).

In each model, we evaluated the effects of the anthropomorphic manipulations of Voice (no voice, TTS voice; sum coded), Pronoun (“I”; “the system”; sum coded) and their interaction. All models also included either Generation or Age as a predictor, which was part of a three-way interaction with Voice and Pronoun. To determine whether sum-coded Generation (Gen Z, Millennial, Gen X, and Baby Boomer), ordered categorical generation (Gen Z < Millennial < Gen X < Baby Boomer), or continuous Age (standardized) best fit the data, we fit three separate models for each dependent variable and retained the model with the lowest corrected Akaike Information Criterion (AICc) using the *MuMIn* package (Bartoń, 2017). We assessed multicollinearity using the *performance* R package (Lüdecke et al., 2021). If the model's variance inflation factor (VIF) exceeded 5, we addressed collinearity as follows: if the source involved an interaction term, the interaction was removed; if it involved multiple correlated predictors, we fit separate models including each predictor individually and used AICc-based model comparisons to determine which predictor to retain.

To evaluate whether effects observed in the retained models could be accounted by other sociodemographic variables, we conducted post hoc sensitivity analyses using models with identical fixed and random effects structures that additionally included Education (5-level ordered factor), Race (white, Black/African American, Asian, Native American; sum coded), Ethnicity (Hispanic, non-Hispanic; sum coded), and Gender (female, male; sum coded) as covariates. We additionally assessed collinearity of these models to determine whether Generation/Age was collinear with any of the other sociodemographic variables and thus accounting for overlapping variance. These analyses were conducted on a subset of the dataset (*n* = 1,475 observations) with complete demographic data and excluding participants reporting gender identities outside the binary male/female categories included in the sum-coded Gender predictor (*n* = 6). Because demographic information was incomplete and unevenly distributed across these variables, these models were treated as post hoc sensitivity analyses rather than primary analyses. Full model outputs for both primary and post hoc analyses are provided in the **Open Science Repository**^5^ for the project (**Tables S1–20**), including 95% confidence intervals.

### 3.1 Anthropomorphism

We calculated each participant's anthropomorphism score (0 to 25) based on their responses to the five Godspeed Questionnaire ratings. The composite measure had good internal consistency (Cronbach's α = 0.84). We modeled anthropomorphism score (centered) with a linear regression. The model including Generation (sum coded) had the lowest AICc (8515.2), while the models with ordered Generation (ΔAICc = 0) did not improve fit, and continuous Age (ΔAICc = +3.0) increased AICc, indicating worse model fit. The retained model, including Generation, showed low collinearity between predictors (all VIF < 5).

**Figure 2:**
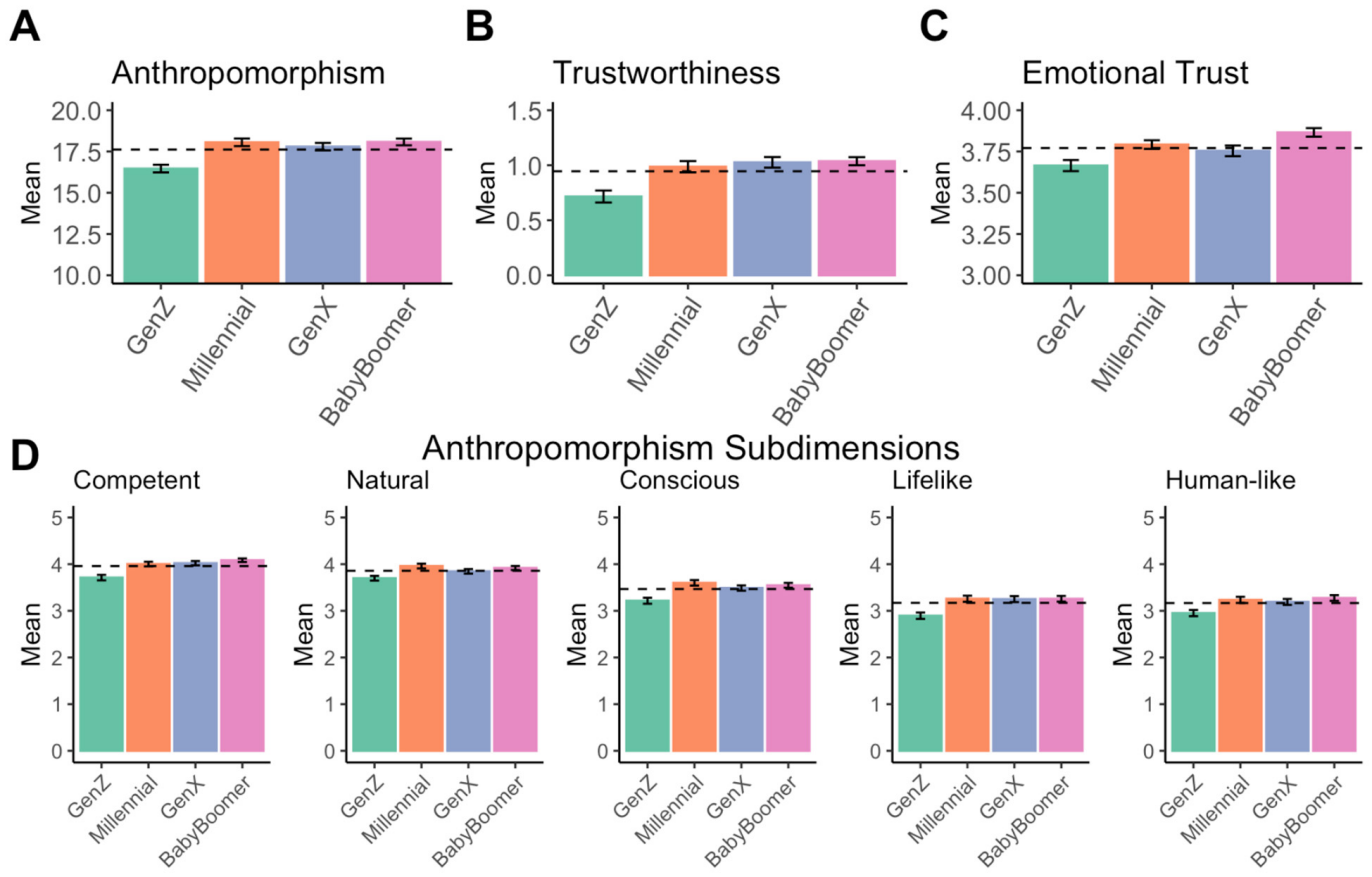
Mean ratings across Generations (from youngest-to-oldest): Generation Z (Gen Z), Millennial, Generation X (Gen X), Baby Boomer. **(A)** Mean anthropomorphism ratings from the Godspeed Questionnaire (overall score 0 to 25), **(B)** Trustworthiness ratings of the LLM (-2 to 2), (**C)** Emotional trust ratings of the LLM (1 to 4) **(D)** Mean ratings for anthropomorphism subdimensions: competent, natural, conscious, lifelike, and human-like from the Godspeed Questionnaire. Error bars indicate standard error of the mean. The dotted lines indicate the grand mean for each rating.

As seen in Figure 2A, the model showed three effects of Generation: Gen Z had lower anthropomorphism scores [Coef = −1.14, SE = 0.2, t = −5.8, p < 0.001], while Millennials had higher scores [Coef = 0.46, SE = 0.19, t = 2.37, p < 0.05], as well as Baby Boomers [Coef = 0.47, SE = 0.19, t = 2.48, p < 0.05] (Gen X did not vary from the grand mean). Additionally, we found that systems that used text + speech had higher anthropomorphism scores [Coef = 0.27, SE = 0.11, t = 2.42, p < 0.05]. There was no difference in anthropomorphism based on Pronoun. No other effects or interactions were observed. A post hoc sensitivity analysis examining additional sociodemographic variables showed low collinearity among predictors (all VIFs < 5). The model (reported in the OSF Repository, Table S1) indicated that the effects for Gen Z and Baby Boomers and Voice remained robust after accounting for demographic covariates. The positive effect observed for Millennials remained directionally similar but was attenuated and no longer statistically significant in this reduced subset analysis.

### 3.2 Trustworthiness

Trustworthiness (untrustworthy ~ trustworthy; Likert responses) was modeled with an ordinal regression with the *ordinal* R package (Christensen, 2015). The trustworthiness model including Generation (sum coded) had the lowest AICc (3690.1), while the models with ordered Generation (ΔAICc = 0) and continuous Age (ΔAICc = +2.9) did not improve fit. The retained model had low collinearity (VIF all < 5).

Summarized raw data is plotted in Figure 2B. As seen in Figure 2B, the trustworthiness model showed that Gen Z rated system as less trustworthy [Coef = −0.45, OR = 0.64, 95% CI [0.54, 0.76], p < 0.001], while Gen X rated is as more trustworthy [Coef = 0.21, OR = 1.24, 95% CI [1.04, 1.46], p < 0.05] (Millennial and Baby Boomer N.S.). The post hoc sensitivity analysis including additional sociodemographic covariates showed low collinearity among predictors (VIF < 5). The primary pattern of results remained robust, with the effects for Gen Z and Gen X remaining significant in the reduced subset analysis (full output provided in OSF Repository, Table S2).

### 3.3 Emotional trust

Emotional Trust was scored (sum of “Yes” responses: “best interest,” “knowledgeable,” “honest,” “unbiased”), but note that the measure had relatively low internal consistency (Cronbach's α = 0.47). We modeled emotional trust with a linear regression. The model with continuous Age (AICc = 2565.83) best fit the data, while sum-coded Generation (ΔAICc = +7.49) and ordered Generation (ΔAICc = +7.49) worsened model fit. The retained model had low collinearity (VIF < 5). As seen in Figure 2C, there was an effect of Age: increasing age was associated with increased emotional trust [Coef = 0.06, SE = 0.01, t = 4.27, p < 0.01]. The post hoc model, including other demographic predictors, also showed low collinearity (VIF < 5) and the effect of Age was still robust.

#### 3.3.1 Subdimensions of emotional trust

We coded whether participants indicated “Yes” (=1) or “No” (=0) for each subdimension of emotional trust and modeled each with a separate logistic regression with the same model structure as the composite Emotional Trust (Age^*^Voice^*^Pronoun). These analyses examined subdimensions of the overall emotional trust score (Section 3.3) and thus p-values were adjusted using the Holm correction procedure. Model outputs are provided in the OSF Repository (**Tables S9–12**).

The best interest model with Age (AICc = 673.39) best fit the model, while sum-coded Generation (ΔAICc = +7.25) and ordered Generation (ΔAICc = +7.25) worsened model fit. The retained model showed low collinearity (VIF < 5), but showed no effects of predictors.

The post hoc model, including other demographic predictors, also showed low collinearity (VIF < 5) and showed no effects.

The knowledgeable model including Age (AICc = 271.44) best fit the data, while sum-coded Generation (ΔAICc = +6.14) and ordered Generation (ΔAICc = +6.14) worsened model fit. The retained model had low collinearity (VIF < 5) also showed no significant effects.

The post hoc model also showed low collinearity (VIF < 5) and no effects.

The honest model including Age (AICc = 622.19) best fit the data, while sum-coded Generation (ΔAICc = +10.82) and ordered Generation (ΔAICc = +10.82) worsened model fit. The retained model showed low collinearity (VIF < 5) and showed an effect of Age: increased age was associated with indicating the system's responses were “honest” [Coef = 0.35, OR = 1.42, 95% CI [1,11, 1.80], p < 0.05]. No other effects were observed.

The post hoc model also showed low collinearity (VIF < 5) but showed no significant effects. The beta coefficient for Age was still in the same direction as the main model.

The unbiased model with sum-coded Generation (AICc = 961.41) best fit the data, while ordered Generation did not improve fit (ΔAICc = +0) and continuous Age worsened fit (ΔAICc = +5.13). The retained model showed low collinearity (VIF < 5) and showed an effect of Generation: Gen Z were less likely to indicate the model was unbiased [Coef = −0.52, OR = 0.59, 95% CI [0.45, 0.79], p = 0.01], while Baby Boomers were more likely to indicate it was unbiased [Coef = 0.78, OR=2.18, 95% CI [1.46, 3.25], p < 0.001]. The post hoc model, including other demographic predictors, had low collinearity (VIF < 5). While the model showed that the Gen Z and Baby Boomer coefficients had the same direction as the main model, neither was significant.

### 3.4 Subdimensions of anthropomorphism

We modeled the five subdimensions of Godspeed Questionnaire (competent, natural, conscious, lifelike, human-like, and Likert responses) in separate ordinal regression models to probe variation across generations. Summarized raw data is plotted in Figure 2D. Because these analyses examined subdimensions of the overall anthropomorphism score (Section 3.1), p-values were adjusted using the Holm correction procedure.

#### 3.4.1 Incompetent ~ competent

The competence model was best fit by the model including Age (continuous) (AICc = 3760.61), while the models with sum-coded Generation (ΔAICc = +4.52) and ordered Generation categories (ΔAICc = +4.52) both worsened model fit. The retained model had low collinearity (VIF < 5). The model showed an effect of Age: higher competence ratings with increased age [Coef = 0.19, OR = 1.20, 95% CI [1.09, 1.32], p < 0.001]. There was also an independent effect of Voice, where hearing a TTS voice led to higher competence ratings, compared to text-only [Coef = 0.12, OR = 1.14, 95% CI [1.03, 1.25], p < 0.05]. No other effects or interactions were observed. The post hoc model including demographic covariates had low collinearity (VIF < 5). While the Age and Voice effects were not significant, their effects remained in the same direction as the main model.

#### 3.4.2 Fake ~ natural

The natural model was best fit including the model with Generation (sum coded) (AICc = 3853.07), while ordered Generation did not improve fit (ΔAICc = 0) and continuous Age worsened fit (ΔAICc = +4.3). The retained model had low collinearity (VIF < 5) and showed effects of Generation: Gen Z rated the system as more fake [Coef = −0.32, OR = 0.73, 95% CI [0.62, 0.86], p < 0.01], while Millennials rated the system as more natural [Coef = 0.25, OR = 1.28, 95% CI [1.08, 1.51], p < 0.05]. No other effects were observed. A post hoc sensitivity model showed low collinearity (VIF < 5). The model coefficients showed the same direction for Gen Z and Millennial in the main model, but were not significant in the sensitivity analysis.

#### 3.4.3 Unconscious ~ conscious

The conscious model with sum-coded Generation best fit the data (AICc = 4351.03), while ordered Generation did not improve model fit (ΔAICc = 0) and continuous Age worsened fit (ΔAICc = +7.2). The retained model had low collinearity (VIF < 5) and showed effects of Generation: Gen Z rated the system as less conscious [Coef = −0.39, OR = 0.68, 95% CI [0.57, 0.79], p < 0.001], while Millennials rated the system as more conscious [Coef = 0.25, OR = 1.28, 95% CI [1.09, 1.51], p < 0.05]. No other effects were observed. The post hoc model including other demographic predictors showed low collinearity (VIF < 5). The model still showed the effect for Gen Z, while the effect for Millennials was no longer significant (though the beta coefficient was still in the same direction).

#### 3.4.4 Artificial ~ lifelike

The lifelike model with continuous Age (AICc = 4615.90) best fit the data, with ordered Generation (ΔAICc = +0.7) and sum-coded Generation (ΔAICc = +0.7) both worsening model fit. The retained model had low collinearity (VIF < 5) and showed an effect of Age: increasing age was associated with rating the system as more lifelike [Coef = 0.15, OR = 1.16, 95% CI [1.06, 1.27], p < 0.05]. No other effects were observed. The post hoc sensitivity model showed low collinearity (VIF < 5). While the model still showed a positive beta coefficient for Age, the model had no significant effects or interactions.

#### 3.4.5 Machine-like ~ human-like

The human-like model with continuous age (AICc = 4566.73) best fit the data, while sum-coded Generation (ΔAICc = +8.53) and ordered Generation (ΔAICc = +8.53) both worsened model fit. The retained model had low collinearity and showed effects of Age: older adults rated the system as more human-like [Coef = 0.15, OR = 1.16, 95% CI [1.06, 1.27], p < 0.05]. Additionally, there was an independent effect of Modality: hearing a TTS voice increased ratings of human-likeness [Coef = 0.14, OR = 1.15, 95% CI [1.05, 1.27], p < 0.05]. No other effects were observed. The post hoc sensitivity model showed low collinearity (VIF < 5). While the model still showed a positive beta coefficient for Age and Voice, the model had no significant effects or interactions.

### 3.5 Qualitative analysis: whether the system “seems like a real person”

To probe whether participants' rationales for anthropomorphism and trust varied by participant age, we analyzed responses to the open-ended question, “Do you think that the system is like a real person? Explain why or why not in a sentence.” Because survey responses are brief, our thematic analysis is not meant as a standalone exploration of participant reasoning (see also: LaDonna et al., 2018), but rather to gain high-level insight into how participants assess human-likeness.

To compare themes across age groups, we focused on participants with relatively low and high anthropomorphism scores. We first identified participants in the lowest (0–15) and highest (21–25) quartiles of anthropomorphism scores. We then randomly selected 100 participants from each generation: 50 from the low-anthropomorphism quartile and 50 from the high-anthropomorphism quartile. Within each quartile, sampling was balanced across the “speech + text” and “text only” conditions (25 participants per condition). This yielded 400 responses for analysis.

As in related work (e.g., Nguyen et al., 2022), four authors on the paper independently coded the responses using thematic analysis (Braun and Clarke, 2006) and each response was validated by a second rater. Based on all participants' responses, we identified four major themes (shown in Table 3): content quality (how accurate) (*n* = 102 responses), conversational style (*n* = 198), entrenched belief (*n* = 106), and knowledge source/access (*n* = 39 responses). Additionally, we identified one sub-theme of conversational style, robotic style (*n* = 79).

**Table 3:** Themes for ‘Does the system seem like a real person? Why or why not.”

Themes	Definition	Examples from dataset
Entrenched beliefs	Beliefs about whether technology can or cannot ever approximate a human, irrespective of the interaction. Often includes anthropodenial (e.g., “it's AI”) and core human attributes (e.g., heart, mind, lived experience, thought, emotion, morality, and belief system).	•*Not really, I feel like you can never imitate a real person* (P158, Gen Z) •*Yes I believe all artificial intelligence have had a human brain at a time* (P175, Gen Z) •*Yes I think the system is like a real person why because it looks like it has a mind of a real person* (P228, Gen X)
Content quality	How well the system did in responding (e.g., accuracy, comprehensiveness, and precision). Includes how competent the system is perceived in providing a high quality response.	•*They answered the question like a real person would and got to the point of the question* (P396, Baby Boomer) •*Yes the information was accurate and had its stats* (P134, Gen Z) •*No because of how general the answers were* (P20, Millennial) •*I think the system is not a real person due to the exact answers* (P128, Gen Z)
Knowledge sources and access	Perceived knowledge base and information access of the system. Includes rationales that the system knows as much or much more than a human.	•*I think the system is smarter than a person* (P233, Gen X) •*No it's more like a search engine* (P310, Baby Boomer)
Conversational style	Response style/tone/sound (figurative and literal) of the system. Often used to describe a robotic style or human-like style of communication.	•*No, I do not think the system is like a real person because it does not have the same dialogue as a real person would. It speaks purely informational with no tones to its voice* (P119, Gen Z) •*Mostly like a person because the responses were conversation like*. (P240, Gen Z)
*Style sub-theme*		
	**Robotic:** Indicated the system did or did not seem robotic or like a robot, machine, computer.	•*No, because it was giving robot sounding answers*. (P109, Gen Z) •*Seemed like a real person. Voice sounded real and not robot like* (P389, Baby Boomer)

We coded the presence of each theme in participants' responses (=1 if present, =0 if not) and modeled responses with a separate logistic regression for each feature from Table 3. Fixed effects included Anthropomorphism Level (low, high; sum coded), Voice Condition (speech + text, text only; sum coded), and their interaction. Factors were sum coded. We assessed collinearity of predictors with the *performance* R package (Lüdecke et al., 2021).

#### 3.5.1 Entrenched beliefs

The model including sum-coded Generation best fit the data (AICc = 464.75), while the model with ordered Generation (ΔAICc = +0) did not improve fit and continuous Age (ΔAICc = +4.81) worsened model fit. The retained model had low collinearity (VIF < 5) and showed effects of Generation: Millennials tended to cite entrenched beliefs more often [Coef = 0.6, OR =1.82, 95% CI [1.25, 2.69], p < 0.01]. For example, as P23, a Millennial, put it, “*The system isn't human [it's] an AI*.” Additionally, there was an effect of Anthropomorphism: individuals with lower anthropomorphism tended to cite entrenched beliefs more often [Coef = 0.33, OR = 1.39, 95% CI [1.09, 1.79], p < 0.01]. There were also two interactions between Generation and Anthropomorphism: Gen Z participants with lower anthropomorphism were more likely to cite entrenched beliefs [Coef = 0.49, OR =1.63, 95% CI [1.03, 2.59], p < 0.05], while Millennials with low anthropomorphism were less likely to [Coef = −0.62, OR = 0.54, 95% CI [0.36, 0.79], p < 0.01]. No other effects or interactions were observed.

The post hoc model including demographic covariates showed low collinearity (VIF < 5). The model showed the same effects for Millennials and Anthropomorphism Category overall, as well as the interactions between Gen Z and Millennials and Anthropomorphism Category. The simple effect of Gen Z was no longer significant, but had the same directionality of the coefficient.

#### 3.5.2 Content quality

The model including continuous Age best fit the data (AICc = 439.99), while the model with sum-coded Generation (ΔAICc = +15.92) and ordered Generation (ΔAICc = +15.92) both worsened model fit. The retained model had low collinearity (VIF < 5). The model showed effects of Age: older adults were less likely to cite content quality [Coef = −0.26, OR = 0.77, 95% CI [0.60, 0.99], p < 0.05]. Additionally, there was an independent effect of Anthropomorphism Category, wherein individuals with lower anthropomorphism were less likely to cite content quality in their rationales [Coef = −0.51, OR = 0.60, 95% CI [0.47, 0.77], p < 0.001]. Put another way, individuals with *higher* anthropomorphism were more likely to cite the content quality. No other effects or interactions were observed. The post hoc including additional demographic covariates showed low collinearity, as well as the same effects of Age and Anthropomorphism.

#### 3.5.3 Knowledge sources/access

**Figure 3:**
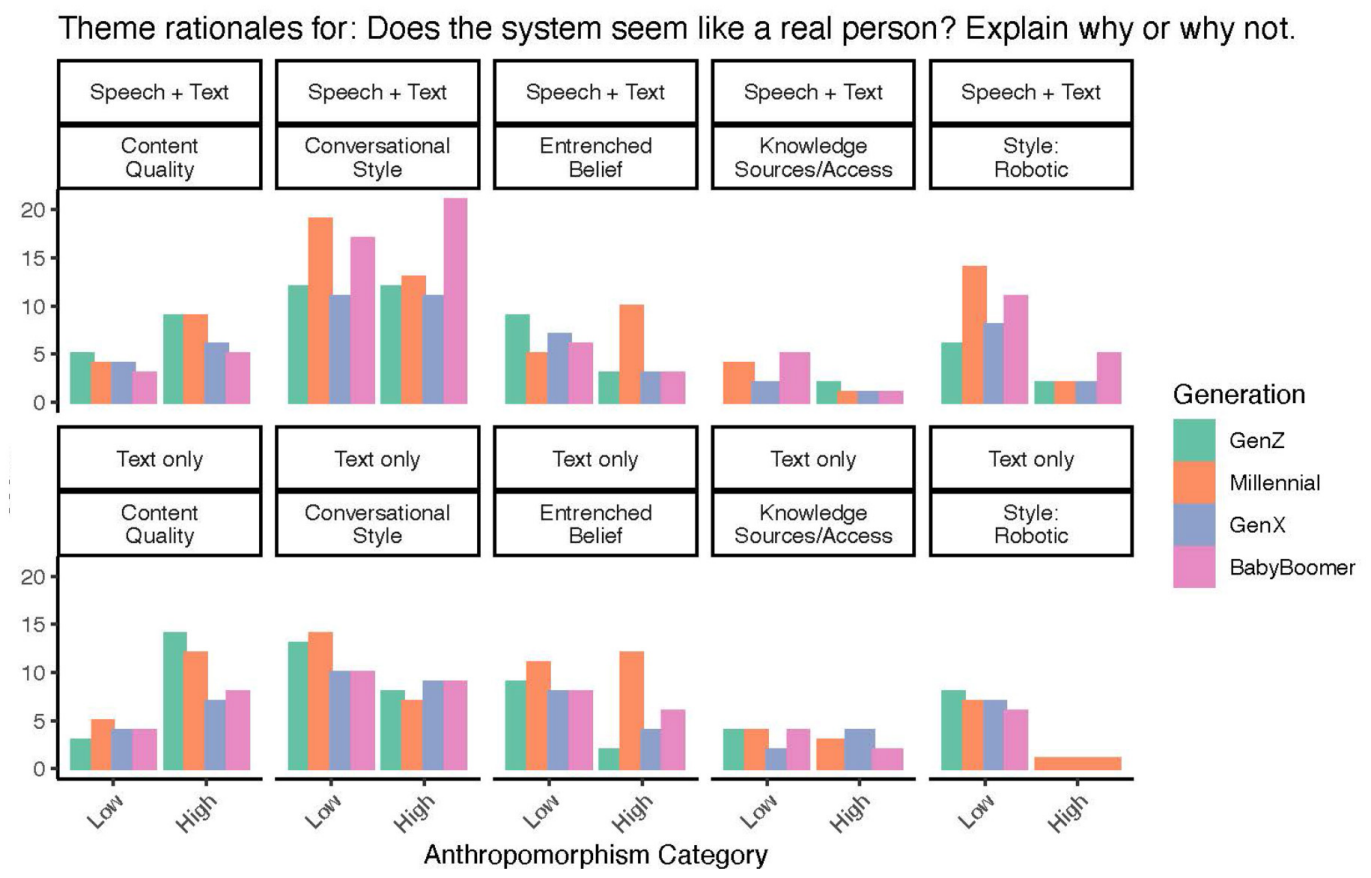
Proportion of responses that included each theme in qualitative responses for “Does the system seem like a real person? Explain why or why not.” across the Generations (Gen Z, Millennial, Gen X, and Baby Boomer) and Anthropomorphism Categories: participants with anthropomorphism scores in the first quartile (“Low”) and fourth quartile (“High”). Plots are faceted by the Voice condition: the top row shows the speech + text condition, while the bottom row shows the text only condition.

The model including continuous Age best fit the data (AICc = 258.65), while the model with sum-coded Generation (ΔAICc = +8.66) and ordered Generation (ΔAICc = +8.66) both worsened model fit. The retained model had low collinearity (VIF < 5). The model showed one three-way interaction between Age, Anthropomorphism, and Voice, where older adults with low anthropomorphism were more likely to cite knowledge sources in their rationales [Coef = 0.42, OR = 1.52, 95% CI [1.04, 2.20], p < 0.05]. Put another way, as seen in Figure 3, older adults with *higher anthropomorphism* in the +voice condition were less to cite knowledge sources and access in their rationales. The post hoc model showed low collinearity (VIF < 5) and showed the same three-way interaction as in the main model.

#### 3.5.4 Conversation style

The model including continuous Age best fit the data (AICc = 558.92), while the model with sum-coded Generation (ΔAICc = +7.43) and ordered Generation (ΔAICc = +7.43) both worsened model fit. The retained model showed low collinearity (VIF < 5). The model showed an effect of Voice: individuals who heard the text-to-speech voice tended to cite conversational style more frequently [Coef = 0.27, OR = 1.31, 95% CI [1.07, 1.60], p < 0.01]. The post hoc model including demographic covariates showed low collinearity (VIF < 5) and the same effect as the main model.

#### 3.5.5 Conversational style subtype: robotic style

The model including continuous Age best fit the data (AICc = 346.08), while the model with sum-coded Generation (ΔAICc = +8.26) and ordered Generation (ΔAICc = +8.26) worsened model fit. The retained model had low collinearity (VIF < 5). While the model showed no difference by Age, there was an effect of Anthropomorphism Category: individuals with lower anthropomorphism were more likely to cite robotic style of speaking more frequently [Coef = 1.37, OR = 3.94, 95% CI [2.20, 6.96], p < 0.001]. Additionally, there was an independent effect of Voice: hearing a text-to-speech voice increased likelihood that participants cited a robotic style [Coef = 0.74, OR = 2.10, 95% CI [1.19, 3.74], p < 0.05].

The post hoc model including demographic covariates also had low collinearity (VIF < 5) and the same effects of Anthropomorphism Category and Voice as in the main model.

### 3.6 Trial-level ratings of trust

#### 3.6.1 Trial-level accuracy ratings

We modeled accuracy ratings with an ordinal mixed effects model with the *ordinal* R package (Christensen, 2015). Fixed effects included Voice, Grammatical Person, Question Context (career, cooking, health, medication, and travel), and all possible interactions. We additionally tested the inclusion of Generation (sum-coded), Generation (ordered), and Age (continuous), with all possible interactions with Voice, Grammatical Person, and Question Context. Random effects included by-Participant and by-Question random intercepts, as well as by-Participant random slopes for Question Context. Factors were sum coded.

The models including Generation (sum-coded and ordered) and continuous Age all showed high collinearity between the interaction of Generation/Age and Question Context (VIF > 5) and thus the interaction was removed. Model comparisons revealed that the model with sum-coded Generation (AIC = 60160.61) best fit the data, with no improvement with ordered Generation (ΔAICc = +0) and a worse fit with continuous Age (ΔAICc = +20.05).

**Figure 4:**
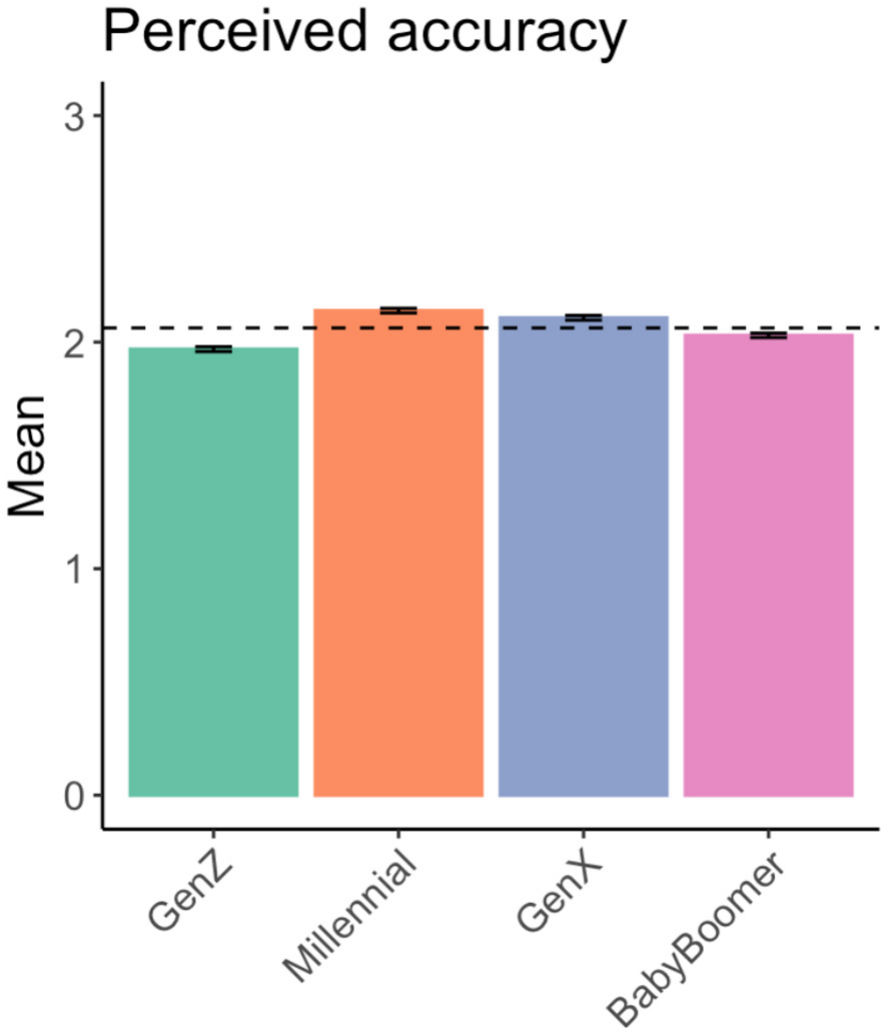
Perceived accuracy of the LLM's output in experimental trials across Generations (not at all accurate = 0, somewhat accurate = 1, mostly accurate =2, completely accurate =3). The dotted line indicates the grand mean, while the error bars indicate the standard error of the mean.

The retained model^6^ had low collinearity (VIF < 5) and showed effects of Generation. As seen in Figure 4, Gen Z rated scores as less accurate [Coef = −0.27, OR =0.76, 95% CI [0.65, 0.89], p < 0.001] while accuracy ratings were higher by Millennial [Coef = 0.32, OR = 1.38, 95% CI [1.19, 1.60], p < 0.001] and Gen X [Coef = 0.18, OR = 1.20, 95% CI [1.03, 1.39], p < 0.05] participants. The releveled model showed lower accuracy for Baby Boomers [Coef = −0.22, OR = 0.80, 95% CI [0.68, 0.93], p < 0.01]. The model also showed an effect of Voice: systems that used text + speech were rated as more accurate [Coef = 0.11, OR = 1.12, 95% CI [1.02, 1.22], p < 0.05]. The post hoc sensitivity model showed the same effects for Generation: less accurate for Gen Z and Baby Boomer, but more for Millennials and Gen X. Additionally, the post hoc model showed the effect of Voice.

#### 3.6.2 Trial-level perceived risk

We modeled risk ratings with a mixed effects ordinal regression, with the fixed effects of Voice, Pronoun, Question Context, and all possible interactions. We additionally tested Generation (sum-coded), Generation (ordered), and Age (standardized) in separate models, with all possible interactions with Voice, Pronoun, and Question Context. We additionally included Accuracy rating (numeric, centered). Random effects included by-Participant and by-Trial random intercepts, as well as by-Participant random slopes for Question Context.

Models that included the interactions of Question Context and Generation/Age were collinear (VIF > 5) and thus the interaction was removed. In the updated models (Age^*^Voice^*^Pronoun + Question_Context + Accuracy), the model with continuous Age (AICc = 66312.83) had the best model fit, relative to the models with sum-coded Generation (ΔAICc = +9.56) and ordered Generation (ΔAICc = +9.55). However, the model including Age showed extremely high collinearity between Age and Voice (VIF = 98.57), while the models including Generation had low collinearity (VIF < 5). As the interaction of an Age/Generation covariate and Voice and Pronoun was part of our main research question, we retained the ordered Generation model.

The retained model showed low collinearity (VIF < 5). While there were no effects of Generation (ordered), Voice, or Pronoun, we did see differences based on Question Context: participants indicated questions about careers were less risky [Coef = −1.17, OR = 0.31, 95% CI [0.14, 0.67], p < 0.01]. We also saw one other effect of Accuracy: if participants rated the response as more accurate, they rated it as less risky as well [Coef = −0.49, OR = 0.61, 95% CI [0.59, 0.63], p < 0.001]. No other effects or interactions were observed. The post hoc model including demographic covariates showed low collinearity (VIF < 5). The same effects of Question Context (career) and Accuracy rating remained significant when accounting for the additional demographic covariates.

#### 3.6.3 Trial-level likelihood to externally validate

We modeled likelihood to validate with external sources with an ordinal mixed effects model, with the predictors of Voice, Pronoun, Question Context, and all possible interactions. We tested inclusion of Generation (sum-coded), Generation (ordered), and Age (continuous) in separate models, with all possible interactions with Voice, Pronoun, and Question Context. Additionally, we included independent fixed effects of Accuracy (numeric, centered) and Risk (numeric, centered). Random effects included by-Participant and by-Trial random intercepts, and by-Participant random slopes for Question Context.

The models including an interaction between Question Context, Voice, and Pronoun were collinear (VIF = 8.86). Thus, the updated model included only the interactions between Generation/Age and Voice and Pronoun and a separate interaction with Generation/Age and Question Context. Model comparisons revealed that the model with continuous Age (AICc = 45049.69) best fit the data, while sum-coded Generation (AICC = + 17.44) and ordered Generation (AICC = + 17.44) both worsened model fit. The retained model had low collinearity (VIF < 5).

The model showed no simple effects of Age, Voice, or Pronoun, but effects of Question Context: participants were less likely to validate for career [Coef = −0.23, OR = 0.79, 95% CI [0.63, 1.00], p < 0.05], cooking [Coef = −0.47, OR = 0.63, 95% CI [0.50, 0.79], p < 0.001], and travel [Coef = −0.29, OR = 0.79, 95% CI [0.59, 0.94], p < 0.05], but more likely to validate for health [Coef = 0.43, OR = 1.54, 95% CI [1.22, 1.95], p < 0.001] and medication [Coef = 0.56, OR = 1.75, 95% CI [1.39, 2.23], p < 0.001].

**Figure 5:**
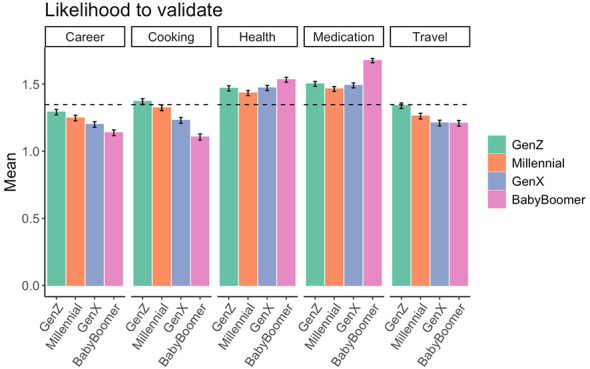
Likelihood of externally validating the system's response (0 = No, 1 = Maybe, 2 = Yes) across Question Contexts and Generations. Error bars indicate standard error of the mean. The dotted line indicates the grand mean.

While there were no simple effects of Age, Age interacted with Question Context. As seen in Figure 5, older adults were less likely to validate for career [Coef = −0.09, OR = 0.91, 95% CI [0.87, 0.96], p < 0.01], cooking [Coef = −0.27, OR = 0.76, 95% CI [0.72, 0.80], p < 0.001] and travel [Coef = −0.12, OR = 0.89, 95% CI [0.84, 0.93], p < 0.001], but more likely to validate for health [Coef = 0.14, OR = 1.15, 95% CI [1.08, 1.22], p < 0.001] and medication [Coef = 0.34, OR = 1.40, 95% CI [1.31, 1.51], p < 0.001].

Additionally, there were independent effects of Accuracy rating: more accurate, less likely they are to validate [Coef = −0.36, OR = 0.70, 95% CI [0.67, 0.73], p < 0.001] and Risk rating: more risky, more likely to validate [Coef = 0.75, OR = 2.12, 95% CI [2.05, 2.20], p < 0.001]. The post hoc model including demographic covariates also showed low collinearity (VIF < 5). The model showed the Question Context effects were still significant for cooking, travel, health, and medication, though the simple effect for career no longer reached significance. The effects for accuracy and risk were also robust. Furthermore, all five Age^*^Question Context interactions remained robust.

## 4 Discussion

This study investigated age- and generation-related differences in anthropomorphism and trust toward a pseudo-LLM system among participants in the United States. In particular, we tested how four age cohorts (Gen Z, Millennials, Gen X, and Baby Boomers) weighed manipulated cues of human-likeness: the presence of a text-to-speech voice (compared to text-only) and the use of the first-person pronoun, “I” (compared to third-person singular, “the system”), on their overall ratings of the system, as well as trial-level ratings for the LLM's response on the dimensions of perceived accuracy, risk, and likelihood to validate with external sources. For each analysis, we compared model fit based on whether age (continuous) or generation (factor) better fit the data. Overall, we observe differences attributed to differences in age and generation that are independent of other sociodemographic effects (as assessed in post hoc sensitivity analyses).

### 4.1 Anthropomorphism

First, we observed generational differences in anthropomorphism, or the attribution of human-like characteristics to the system (Epley et al., 2007; Waytz et al., 2010). Gen Z (youngest age group) showed the lowest anthropomorphism ratings, while Baby Boomers (oldest age group) had higher anthropomorphism scores on average. These results are consistent with prior work showing that older adults perceive voice technology, such as voice assistants and TTS voices, as more human-like than younger adults (Herrmann, 2023; Zellou et al., 2021). At the same time, we did not see a linear increase of anthropomorphism with increasing generation (Gen Z < Millennial < Gen X < Baby Boomer). Rather, Millennials differed from Gen Z and Gen X differed from Baby Boomers. This finding is consistent with generational cohort theory, wherein generations may capture differences in shared sociotechnical experiences (Okros, 2020; Twenge et al., 2015). Notably, while generation-related differences emerged for the composite anthropomorphism score, derived from the Godspeed Questionnaire, we only saw one subdimension that was robust to the sensitivity analysis: Gen Z rated the LLM as less conscious. While the overall pattern was similar across other subdimensions in the main analysis, with Gen Z showing lower ratings of competence, naturalness, lifelikeness, human-likeness of the LLM, these effects were not robust in the sensitivity analyses. This pattern suggests that Gen Z's differences in anthropomorphism might be more driven by perceptions of consciousness than by broader anthropomorphic perceptions, though more work is needed to test this possibility.

To further probe age- and generation-related differences in anthropomorphism, we conducted a qualitative analysis of a subset of the data to probe whether participants varied in their rationales as to whether “the system seems like a real person or not.” We identified four main themes that shaped these responses: content quality (e.g., perceived accuracy), conversational style, entrenched beliefs, and knowledge source/access. Additionally, we identified one sub-theme of conversational style, robotic style, when participants indicated a robotic, mechanical, or machine-like style of communication. Some of these themes are similar to those in related work. For example, in a meta-analysis of 29 articles, Rheu et al. (2021) also found that the style of interaction shaped trust, including voice characteristics.

We also observed some generational differences in rationales: Millennials tended to cite entrenched beliefs for human-likeness (e.g., “*No because it's not*” P22, Millennial). While Millennials with lower anthropomorphism tended to cite entrenched beliefs less often, we found that Gen Z with lower anthropomorphism cited them more often (e.g., “*No, robots and AI will never be conscious like the children of God, Jesus has made”* P160, Gen Z). For other rationales, we observed more variation by age; for example, older adults were less likely to cite content quality than younger adults. Overall, these findings suggest that younger and older adults may attend to different types of information when evaluating the human-likeness of AI systems.

### 4.2 Ratings of trust

Additionally, participants varied by generation in how they rated the system's overall trustworthiness. While Gen Z rated the systems as less trustworthy, Gen X rated the system as *more* trustworthy. When examining trial-level ratings of perceived accuracy of the pseudo-LLM's answer, we additionally observed generational differences: Gen Z tended to rate the system's responses as less accurate. Gen Z's lower trust ratings parallel prior work showing weaker trust for Gen Z, even relative to Millennials (Primanto and Amin, 2024). While some work has shown stronger trust for younger generations (e.g., in voice assistants in Noah and Sethumadhavan, 2019; in ChatGPT in Chan and Lee, 2023), we instead see that the younger adults in our study appear to be more wary and distrustful of information from a LLM. This pattern is consistent with Vinichenko et al. (2022), who found that Slovakian and Russian young adults were more wary of historical information generated by an AI system. One possible explanation is that younger adults, who have greater exposure to contemporary AI systems, may be more attuned to concerns about inaccuracy, bias, and hallucinations. Consistent with this interpretation, Gen Z participants attributed fewer human-like qualities to the system and were more likely to invoke pre-existing beliefs about AI when evaluating it.

Paralleling Gen Z, we observed that Baby Boomers also rated information the pseudo-LLM presented was less accurate. This was a somewhat unexpected finding as it does not reflect a simple increase or decrease across generations. Notably, Baby Boomers showed *stronger* anthropomorphism of the systems than younger cohorts, yet they did not show correspondingly higher perceptions of accuracy. This pattern suggests that anthropomorphism and trust, while related, may be partially dissociable. Prior work has shown that more human-like systems are more trustworthy (Hancock et al., 2011; Rheu et al., 2021; Waytz et al., 2014). However, our findings indicate that attributing human-like qualities to a system does not necessarily translate into greater confidence in its outputs. One possibility is that different generations rely on distinct evaluative processes when interacting with AI systems. Baby Boomers may perceive the system as more human-like while nevertheless maintaining caution about the reliability of information provided by technology. Older adults are more accustomed to receiving information from established institutional sources, such as newspapers and television news, while younger adults increasingly learn information from digital and social media platforms; recent survey data indicate that Gen Z reports lower trust in national and local news organizations than older generations, while exhibiting comparatively higher trust in social media sources (Shearer, 2025). The position of LLMs within this information landscape remains ambiguous. Unlike traditional news organizations, LLMs do not represent a clearly identifiable institution. Consequently, different generations may evaluate LLM outputs using distinct expectations regarding source credibility and information source.

As all information in the present study was intentionally selected to be accurate and unbiased, lower perceived accuracy ratings among Gen Z and Baby Boomers suggest a greater degree of skepticism toward LLM-generated information than would be expected based on the quality of the content itself. While we did not manipulate accuracy in the present study, these findings suggest that evaluations of LLM outputs may be influenced not only by the content provided, but also by pre-existing beliefs about the reliability of AI systems.

While there was no difference by age or generation in trial-level ratings of risk, participants did show age-based differences in their likelihood to externally validate the information across contexts. Older adults were more likely to validate information about health and medication, while younger adults were less likely to validate clinical information. Conversely, younger adults were more likely to validate information about cooking, travel, and careers, while older adults were less likely to look up this information. Here, one possibility is that adults over age 60 are likely to have at least one health disorder (Ward and Schiller, 2013) and take multiple medications (Charlesworth et al., 2015), which could make trusting information about these domains as riskier for older adults.

Taken together, the age and generational differences for trust observed in the present study can help explain the disparate results in the prior literature, where sometimes younger adults show stronger trust toward technology (Noah and Sethumadhavan, 2019), but other cases where older adults show stronger trust (Pak et al., 2014, 2017). Here, we show that question context is an important factor, as well as the type of rating: if rating the overall trustworthiness of the system or subdimensions of trust (e.g., accuracy, risk, and validation).

Furthermore, while our modeling of emotional trust (Benbasat and Wang, 2005; Wang and Benbasat, 2007) of the system revealed differences by age, with older adults showing greater emotional trust of the pseudo-LLM, we note that this summed emotional trust measure had poor internal consistency (Cronbach's α = 0.47). We additionally examined each constituent item of emotional trust (system is unbiased, knowledgeable, honest, responds in my best interest), but the age-based effects did not consistently emerge across these subdimensions. Therefore, these findings should be interpreted with caution. Future work using other scales of emotional trust might better reveal potential differences.

### 4.3 Effect of anthropomorphic manipulations

In addition to testing age and generational differences in anthropomorphism and trust overall, we also investigated whether age/generation would guide how participants would integrate the manipulated cues of human-likeness in the current study: the presence of a human-like TTS voice and use of the first-person pronoun, “I.” Here, we saw that all ages were sensitive to the presence of a text-to-speech voice. Anthropomorphism increased across generations in the presence of a TTS voice compared to text-only. Furthermore, hearing a voice led participants to rate the information the LLM presented as more accurate. Both of these findings paralleled our results from the larger dataset that did not investigate differences by age (Cohn et al., 2024).

We only saw one limited effect where age mediated the influence of the TTS voice: in our qualitative analysis, older adults with higher anthropomorphism in the TTS condition were less to cite knowledge sources and access in their rationales, compared to text-only. Here, one possibility is that the strength of the TTS voice varies by age in rationales for what makes a system “seem” like a real person. At the same time, the qualitative analysis was on a subset of the overall dataset (only *n* = 400) and the knowledge access/source theme was the least commonly identified theme, occurring in just 9.75% of responses, making this interpretation more tenuous.

While we predicted potential age/generational effects based on the use of the first-person pronoun (“I”), we did not observe differences in the current study. A lack of a difference based on person-wording parallels related work, wherein participants did not show differences in trust based on a more human-like explanation in wording compared to a more succinct response (Kahr et al., 2023). One interpretation is that specific wording differences might not shape anthropomorphism and trust; but it is also possible that participants were ignoring the repeated preamble (e.g., “Here's what I found…” | “Here's what the system found…”) as it was redundant in each trial.

### 4.4 Limitations and future directions

The current study has several limitations that can serve as directions for future research. First, all of the information in the system's responses was accurate, to the best of our knowledge. As such, we see that trust and accuracy ratings are also relatively high. This also parallels Kahr et al. (2023) who found increased trust in high accuracy models. Related work has explored the effect of errors (Kahr et al., 2024) and, in the case of autonomous vehicles, risky behavior (Rovira et al., 2019) to probe trust calibration. Future work testing the effect of inaccurate and/or biased information can further probe differences in trust calibration across generations, as well as in relation to varying system reliability and error production.

Second, while all participants had prior experience with voice technology, the experiment did not assess each individual's experience and attitudes about technology and the Internet (Blank and Dutton, 2012). In addition to analyzing participants' technology exposure and attitudes, future work can deepen our exploration by analyzing participants' literacy within the domains of interaction (e.g., medical literacy). Third, the study used predetermined questions and responses with a pseudo-LLM, each lasting a single turn, which could reduce each individual's intrinsic motivations to engage with responses. We encourage scholarship to extend this work to naturalistic LLM use cases where users bring real intents.

Additionally, several participants indicated the need for follow-up dialogue in their interactions with the system during the study. In practice, users often engage in multi-turn conversations in which systems retain information from prior turns and provide clarification or repair when misunderstandings occur (Cuadra et al., 2021). Future work could explore anthropomorphism ratings of multi-turn conversations on the same topics and question types, but in contexts relevant to individuals.

Fourth, while we did observe some limited age- and generation-based variation in the qualitative responses for why the system “seems like a real person or not,” participants had limited space for their response. Future work exploring more in-depth explanations, such as in interviews, could shed further insight to the sources of generational differences in rationales (e.g., entrenched belief, conversational style, etc.) and their interactions. Fifth, the experiment manipulated two anthropomorphic cues: the presence of a TTS voice (compared to no voice) and use of the first-person (“I”). As the repetitive “Here's what I found | the system found” may have led participants to ignore the preamble, we encourage future work to explore the impact of more varied linguistic cues, such as other referential terms, on the degree to which users anthropomorphize LLMs and in more naturalistic conversational settings. Other work has shown other types of anthropomorphic manipulations shape trust in systems as well (e.g., facial features in social robots; Ghazali et al., 2018). The extent to which multimodal anthropomorphic cues might interact (e.g., in voice, visual expression) to shape trust in an LLM is an open question. Additionally, we acknowledge that this study did not explore the sociolinguistic impact of the TTS voice and we used a singular voice here. Future research could benefit from probing additional voices, such as those varying in apparent age and gender, as well as the manipulation of isolated fine grained phonetic details to uncover the role of sociolinguistic variation in anthropomorphism. Finally, we acknowledge that this study centered the experiences of U.S. based users of LLM technology and experiences with such technology may vary cross-culturally. Future work should explore multicultural experiences with LLMs to understand how anthropomorphism varies cross-culturally and cross-linguistically.

## 5 Conclusion

Overall, this study highlights the importance of considering age and generation when examining how people perceive and trust large language models. As LLMs become increasingly embedded in different parts of everyday life, understanding how different groups perceive and respond to these systems will become increasingly important. Our findings suggest that anthropomorphism and trust are shaped not only by the characteristics of the system itself, but also the expectations and beliefs users bring to the interaction. More broadly, these results underscore the role of social-cognitive processes in shaping how people evaluate and engage with AI systems.

## Data Availability

The datasets presented in this article are not readily available because the data is proprietary to Google Research. Requests for access should be directed to the corresponding author. Requests to access the datasets should be directed to mdcohn@ucdavis.edu.

## Footnotes

1
https://chat.openai.com/

2
https://gemini.google.com/

3 https://doi.org/10.17605/OSF.IO/5WU97)

4
https://cloud.google.com/text-to-speech/docs/voices

5
https://doi.org/10.17605/OSF.IO/5WU97

6 Generation^*^Voice^*^Pronoun + Question(1+ Question| Participant) + (1| Trial).
