# Supplementary material for: Age and generational differences in anthropomorphism and trust in large language models
Source: Front Cognit. 2026 Jul 20;5:1824836. doi: 10.3389/fcogn.2026.1824836 (PMC13430600; doi:10.3389/fcogn.2026.1824836)
Supplement: Supplementary file 1 [file Table_1.pdf]

Supplementary Table 1.

Question Context	Question	System Response	Duration (s)
Medication	What's the dosage for the medication, Levaquin?	Here's what [I | the system] found: depending on the type of infection, the usual dose of Levaquin is 250 mg given once daily for 5 days.	10,000
Medication	Does the medication Corgard have any dangerous side effects?	Here's what [I | the system] found: side effects for Corgard include numbness in your hands or feet, dizziness, feeling tired, and upset stomach.	7,000
Medication	Should I take the medication Topamax if I'm already taking Tylenol?	Here's what [I | the system] found: there are no interactions reported between Topamax and Tylenol. However, this does not necessarily mean no interactions exist. Always consult your healthcare provider.	11,000
Medication	What's the cost for the medication, Relenza?	Here's what [I | the system] found: depending on the pharmacy you visit, the cost for Relenza inhalation powder could be around $72 for a supply of 20 mg.	10,000
Health	What are symptoms of stroke?	Here's what [I | the system] found: common symptoms of stroke include sudden weakness on one side of the body, vision problems, and sudden difficulty speaking.	8,000
Health	Is cancer hereditary?	Here's what [I | the system] found: up to 10% of all cancers may be caused by inherited genetic changes, meaning your risk of getting cancer may be increased.	8,000
Health	What should I do to reduce my risk of diabetes?	Here's what [I | the system] found: to reduce your risk of diabetes, you should eat a balanced diet and exercise regularly.	6,000
Health	If my dad has heart disease, what are the chances I'll get it too?	Here's what [I | the system] found: if one of your immediate family members had a heart attack, stroke, or heart disease before age 60, this may indicate a family history of premature heart disease.	12,000
Career	How commonly do people switch jobs into healthcare?	Here's what [I | the system] found: it is not as common for people to switch jobs into medicine as it is for other fields, such as computer science.	7,000
Career	What are the job-related health risks in finance?	Here's what [I | the system] found: some risks in finance include chronic stress due to demanding workloads, tight deadlines, and making important financial decisions.	8,000
Career	Would I earn enough as an educational administrator?	Here's what [I | the system] found: the average pay range for a Higher Education Administrator varies greatly (as much as $55,816), suggesting there may be many opportunities for advancement.	12,000
Career	How many years of training would I need to get into computer science?	Here's what [I | the system] found: a bachelor's program in Computer Science generally takes four years to complete, while a master's degree may take an additional one to two years.	10,000
Travel	Where is the world's tallest tree?	Here's what [I | the system] found: the world's tallest tree is Hyperion, which is 344 feet tall and located in Redwood National Park in California, United States.	10,000
Travel	What are the risks in going in a cave?	Here's what [I | the system] found: the most common causes of caving accidents include falling, being struck by falling objects, and hypothermia.	7,000
Travel	Should I visit the world's tallest volcano on a vacation?	Here's what [I | the system] found: Mauna Loa is a massive shield volcano. At 13,680 ft above sea level, hiking to its summit requires a good level of physical fitness and endurance.	11,000
Travel	How long would it take to reach the top of the world's tallest mountain?	Here's what [I | the system] found: from base camp, it takes about 40 days to reach the summit of Mount Everest at 29,029 ft.	7,000
Cooking	What temperature do I need to cook chicken to?	Here's what [I | the system] found: all poultry should reach a safe minimum internal temperature of 165 °F.	7,000
Cooking	Can I drink milk that's been expired for 2 days?	Here's what [I | the system] found: unopened milk generally stays good for 5-7 days past its listed date if stored properly, while opened milk lasts at least 2-3 days past this date.	10,000
Cooking	If I have an allergy to peanuts, should I eat peanut oil?	Here's what [I | the system] found: peanut oil is typically safe because it is highly refined and has almost no detectable peanut protein.	11,000
Cooking	How much is a dash of salt?	Here's what [I | the system] found: a dash is somewhere between 1/16 and 1/8 teaspoon.	4,000
